# Architecturally Mediated Allostasis and Neurosustainability: A Proposed Theoretical Framework for the Impact of the Built Environment on Neurocognitive Health

**DOI:** 10.3390/brainsci15020201

**Published:** 2025-02-15

**Authors:** Cleo Valentine, Heather Mitcheltree, Isabelle A. K. Sjövall, Mohamed Hesham Khalil

**Affiliations:** 1 Department of Architecture, University of Cambridge, Cambridge CB2 1PX, UK; hdm30@cam.ac.uk (H.M.); mhmhk2@cam.ac.uk (M.H.K.); 2 Institute of Behavioural Neuroscience, University College London, London WC1E 6BT, UK; isabelle.sjovall.22@ucl.ac.uk

**Keywords:** allostasis, architecture and mental health, physiological stress, environmental neuroscience, neurosustainability, neuroarchitecture

## Abstract

The global rise in mental health-related disorders represents a significant health and wellbeing challenge, imposing a substantial social and economic burden on individuals, communities, and healthcare systems. According to the World Health Organization, one in four people globally will be affected by mental or neurological disorders at some point in their lives, highlighting a significant global health concern that warrants carefully considered and innovative responses. While mental health challenges arise from complex, multifaceted factors, emerging research indicates that the built environment—the architecture of our homes, workplaces, and public spaces—may exert a critical but underappreciated influence on mental health outcomes. This paper outlines a novel theoretical framework for how visual stressors in the built environment might trigger neurophysiological stress responses via the HPA and SAM axes, potentially contributing over time to allostatic load. In this paper, it is proposed that chronic physiological strain can alter neuroplastic processes and neurogenesis in key brain regions—such as the hippocampus, prefrontal cortex (PFC), anterior cingulate cortex (ACC), and amygdala—thereby affecting cognitive health, emotional regulation, and overall mental wellbeing. Drawing on the principle of neurosustainability, this paper suggests that long-term exposure to stress-inducing environments may create feedback loops, particularly involving the amygdala, that have downstream effects on other brain areas and may be linked to adverse mental health outcomes such as depression. By presenting this framework, this paper aims to inspire further inquiry and applied experimental research into the intersection of neurophysiology, mental health, and the built environment, with a particular emphasis on rigorous testing and validation of the proposed mechanisms, that may then be translated into practical architectural design strategies for supporting health and wellbeing. In doing so, it is hoped that this work may contribute to a more holistic approach to improving mental health that integrates the creation of nurturing, resilient spaces into the broader public health agenda.

## 1. Introduction

Mental health is a key factor for overall health and quality of life [1]. The global rise in mental health-related disorders represents a significant health and wellbeing challenge to individuals, communities, and societies worldwide. According to the World Health Organization, one in four people will be affected by mental health or neurological disorders at some point in their lives [2], and recent estimates suggest that more than 970 million individuals worldwide live with a mental health disorder [3]. Mental health can be described as the absence of mental disorders (for example, depression, generalized anxiety, schizophrenia, and bipolar disorder) and the ability to cope with normal stresses of life, work productively, and contribute to society [4].

Neurological disorders are conditions that impact how the nervous system functions, and may affect cognition, emotions, and movement [5]. The consequences are profound—mental health conditions represent a leading cause of disability, contributing substantially to the global disease burden, reduced economic productivity, and increased healthcare costs [3,6]. The societal toll includes not only financial strain but also pervasive stigma, social exclusion, and diminished quality of life for those affected and their families.

In recognition of the need for extensive and coordinated change, the World Health Organization’s 2022 report, *World Mental Health Report: Transforming Mental Health for All*, calls for collective action and a transformative approach to promoting and protecting mental health [3]. Part of this transformative approach needs to be a comprehensive examination of factors beyond traditional healthcare interventions, including an often-overlooked dimension, the built environment. The places in which we live, work, learn, and interact influence psychological wellbeing, cognitive functioning, and stress physiology. By expanding our focus to encompass how physical spaces may exacerbate or ameliorate mental health conditions, new avenues are opened for prevention, intervention, and policy innovation [7].

In response to these public health concerns, the concept of neurosustainability offers a multidisciplinary framework that integrates neuroscience, psychology, and environmental studies to promote better mental health and cognitive functioning. This framework posits that the environments we inhabit—both natural and built—play a critical role in shaping our neural architecture and psychological wellbeing. By understanding and optimizing the environmental factors that influence neurophysiological health, neurosustainability aims to enhance cognitive performance and mitigate the impact of adverse built environment design elements on mental health disorders. However, to date, research within the field of neuroarchitecture has not fully considered the influence of architecturally mediated allostatic load—prolonged physiological stress responses elicited by exposure to the built environment—on neurological processes and neurosustainability. This paper develops a theoretical framework for the interrelationship between architecturally mediated allostatic overload, neurosustainability, and mental health, with a particular focus on three key brain regions: the amygdala, hippocampus, an prefrontal cortex (PFC). In doing so, this paper establishes testable hypotheses for future empirical investigations into how architectural design choices may modulate neurobiological stress responses and cognitive function.

Environmental factors, particularly those associated with the built environment, have been increasingly recognized for their impact on health outcomes. The field of environmental psychology explores the dynamic interplay between individuals and their surroundings, emphasizing how physical spaces can influence emotions, behaviors, and physiological states. Architectural design, as a fundamental component of the built environment, has the potential to either mitigate or exacerbate stress responses. Features such as lighting, spatial layout, acoustics, and access to natural elements can significantly influence psychological wellbeing and neurological functioning [8,9,10,11,12,13,14,15].

Despite the acknowledged importance of environmental factors, there are significant gaps in research in terms of the interrelationship between the design of the built environment and neurophysiological health. Methodological limitations and the complexity of spatial variables have hindered comprehensive investigations into how the design of the built environment contributes to neurophysiological stress responses and mental health outcomes [16]. Moreover, traditional approaches have often overlooked the nuanced ways in which architecture and spatial complexity can affect neurogenesis (the creation of new neurons) and neuroplasticity (the brain’s ability to reorganize itself by forming new neural connections) [17,18,19].

**Figure 1:**
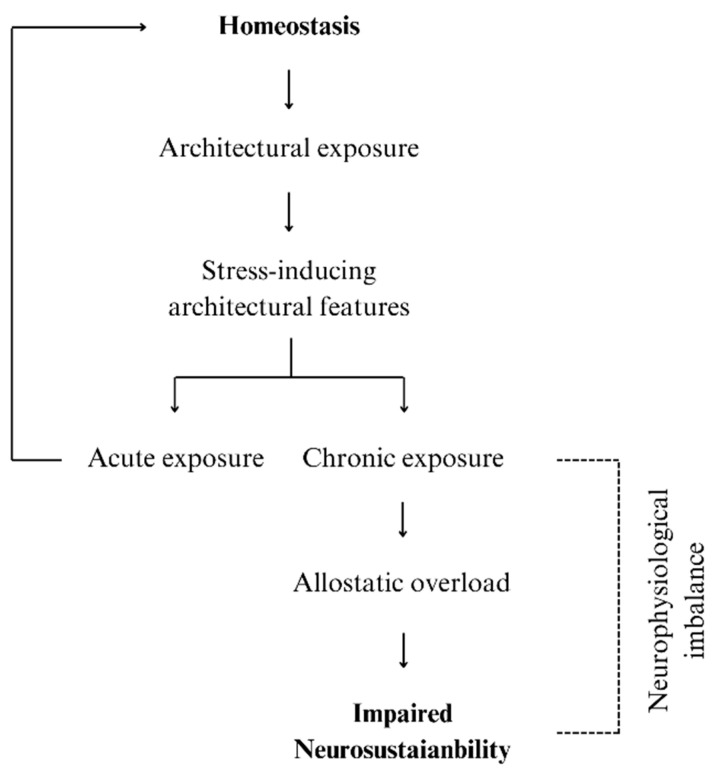
The relationship between homeostasis, chronic exposure to stress inducing architectural features, and neurophysiological imbalance leading to impaired neurosustainability.

The purpose of this paper is to bridge these gaps by exploring how architecturally mediated neurophysiological stress responses influence neurogenesis, neuroplasticity, and ultimately, mental health. Through an interdisciplinary lens that integrates neuroscience, environmental psychology, and consideration of the impact of the design of the built environment, this paper outlines potential mechanisms and pathways by which chronic exposure to stress-inducing built environment elements may lead to sustained activation of stress-related systems, contribute to allostatic load, and impair fundamental processes of brain adaptability and plasticity (see Figure 1). By examining the potential influence of these neurobiological changes on mental health, we can begin to understand how specific design strategies might impact mental health challenges.

This work advocates for a paradigm shift in how spaces are conceptualized and designed and calls for design consideration of neuroarchitecture and neurosustainability. By grounding architectural decisions in an evidence-based understanding of neural processes, it may be possible to create environments that support mental wellbeing, reduce the global burden of mental health disorders, and foster healthier, more resilient communities. This paper presents a theoretical foundation, and in doing so sets the stage for future empirical work aimed at informing architects, urban planners, policymakers, and public health professionals about the critical intersection of built environment design and mental health.

## 2. Physiological Stress Responses and Allostasis

Physiological stress responses encompass the body’s complex reactions to perceived or actual threats to homeostasis—a state of balanced functioning across physiological systems [20]. These threats, known as stressors, disrupt homeostasis and initiate a cascade of physiological and behavioral adaptations designed to restore equilibrium [20]. The stress response is a fundamental adaptive mechanism that helps organisms maintain their internal equilibrium and broader survival functions by enabling them to confront and manage potentially threatening challenges [21]. This intricate response is mediated by a coordinated interplay among the nervous, endocrine, and immune systems, and involves the activation of the sympathetic–adreno-medullary (SAM) axis, the hypothalamic–pituitary–adrenal (HPA) axis, and associated immune pathways [22].

Upon encountering a stressor, sensory information is processed by the amygdala [23]. The amygdala communicates with the hypothalamus, particularly the paraventricular nucleus (PVN), to initiate the stress response. The SAM axis represents the rapid response system, often referred to as the “fight or flight” response. Activation of the PVN stimulates preganglionic sympathetic neurons in the spinal cord, which innervate the adrenal medulla. The adrenal medulla secretes catecholamines—primarily adrenaline (epinephrine) and noradrenaline (norepinephrine)—into the bloodstream [24]. These catecholamines bind to adrenergic receptors on target organs, inducing physiological changes such as increased heart rate, vasoconstriction, bronchodilation, and enhanced metabolic activity to supply energy substrates [25].

**Figure 2:**
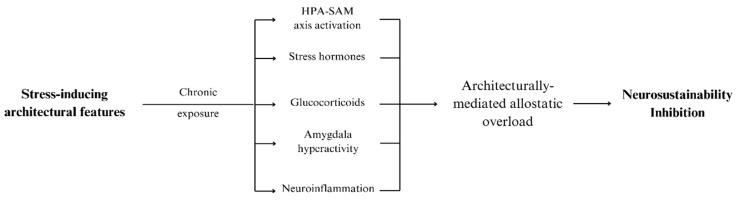
The relationship between stress-inducing architectural features, activation of the HPA-SAM axes, and neurosustainability inhibition.

In parallel, the HPA axis mediates a slower but sustained hormonal response to stress. The PVN secretes corticotropin-releasing hormone (CRH) and arginine vasopressin (AVP) into the hypophyseal portal system, which carries them to the anterior pituitary gland. CRH and AVP stimulate the release of adrenocorticotropic hormone (ACTH) from corticotroph cells [26]. ACTH then travels via the bloodstream to the adrenal cortex, where it stimulates the synthesis and release of glucocorticoids, primarily cortisol in humans [27]. Cortisol exerts widespread effects across various bodily systems, including promoting gluconeogenesis, lipolysis, and protein catabolism to increase glucose availability [28]. Cortisol modulates immune function by exerting anti-inflammatory effects and provides negative feedback to the hypothalamus and pituitary to regulate the stress response [29]. The SAM and HPA axes are interconnected. The initial rapid response of the SAM axis can modulate HPA axis activity, and prolonged activation of the HPA axis can influence the baseline activity of the sympathetic nervous system, which regulates functions such as heart rate, blood pressure, and vascular resistance [30]. This integrated response has evolved to facilitate appropriate physiological adaptations to various stressors [31]. The relationship between stress-inducing architectural features, activation of the HPA-SAM axes, and neurosustainability inhibition is illustrated in Figure 2.

### 2.1. Architectural Design as a Mediator of Stress Responses

Stress can be elicited by a range of environmental, psychological, and biological factors [32]. Environmental stressors are characterized by the strain induced by unfavorable or challenging external conditions [32]. Examples include exposure to excessive noise, pollution, overcrowding, unsafe living environments, and, as emerging research suggests, aspects of the design of the built environment. Chronic exposure to such stressors can lead to persistent activation of stress response systems. Psychological stressors originate from cognitive or emotional challenges, including perceived threats, trauma, or negative self-perceptions [32], whereas biological stressors refer to factors that disrupt the body’s internal balance, such as illness, injury, lack of sleep, or poor nutrition [32]. Despite their distinct origins, these stress categories converge on common and integrated physiological pathways to produce physiological stress responses [22]. The design of the built environment profoundly impacts physiological and psychological wellbeing by mediating stress responses and influencing an individual’s perception of their surroundings. Understanding how the built environment affects stress is essential for creating spaces that promote health and reduce the risks associated with chronic stress.

#### 2.1.1. Architectural Forms

Research in neuroarchitecture suggests that the design and arrangement of the built environment impact physiological stress responses. Advances in virtual reality (VR) and clinical biosensors have enabled more precise measurement of physiological changes in response to architectural and built environment stimuli. In a review of the physiological effects of architectural forms, Valentine [33] found evidence that features such as curvature, proportion, and enclosure significantly influence stress responses across a range of settings. This scholarship builds upon Palmer’s [34] foundational work, which connected the built environment’s influence to underlying visual processes. Palmer proposed that cells in the visual cortex respond to variations in size or scale, suggesting that the brain’s interpretation of spatial cues may shape how individuals experience architectural environments. While distances from and the spatial dimensions of the environmental milieu relative to the body are important considerations [9], it remains unclear whether any one dimension (i.e., ceiling height, window size) impacts human neurophysiology more than others.

Understanding the impact of the design of the built environment on inhabitants is challenging due to the interconnected nature of different aspects of spatial environments. For example, enclosure levels—affected by window size and placement—directly influence daylight access, which in turn affects reliance on artificial lighting. Similarly, materials, color, viewing distance, and aspect ratio are intertwined with how geometric forms are perceived. Since individuals experience spaces dynamically, isolating specific built environment features requires careful formulation of research methodologies and an appreciation of the complex ways in which form, along with other features, shapes human experience of the environmental milieu.

##### Curvature

Curvature refers to the rate at which a curve changes direction. Research examining the impact of the visual characteristics of transitional spaces on the stress recovery of participants (*n* = 40) exposed to different VR environments, conducted by Li et al. [35], found that curved environmental features were more effective for stress recovery after participant exposure to the Trier Social Stress Test (TSST). The research used a range of physiological data such as heart rate and blood pressure, along with psychological measures (State–Trait Anxiety Inventory test) to assess the impact of the visual characteristics of the environment on stress recovery. Similarly, Shemesh et al. [36] assessed stress responses to 27 virtual scenes among 112 participants (*n* = 112) and found an inverse relationship between the degree of wall curvature and stress, as evidenced by larger pupil diameter and higher galvanic skin response. In a small study that examined EEG responses in 17 participants (*n* = 17), Banaei et al. [12] found that greater curvature elicited enhanced pleasure and emotional arousal, as indicated by increased theta wave activity. Strachan-Regan and Baumann (2024) used virtual reality to evaluate how room shape (curved vs. rectangular) affects mood, creativity, and physiological response in a virtual reality experiment (*n* = 35) [37]. Using a within-subject design, participants experienced both room types while researchers measured positive/negative affect (via the PANAS questionnaire), heart rate (via pulse oximeter), and creativity (via Guilford Alternative Uses Task). The study found that the curved room resulted in higher positive affect, lower heart rate, and better creative performance compared to the rectangular room. While these studies offer valuable insights, caution should be exercised in generalizing findings due to the field’s emerging nature and the specific scopes and methodological limitations of the studies to date.

##### Enclosure and Proportion

Enclosure, related to window configuration and dimensions, is closely linked with proportion. Emerging research suggests that increased enclosure is associated with higher physiological stress responses, while reduced enclosure is associated with decreased stress [11,13,14]. Fich et al. [11], in a study examining cortisol levels among 49 male participants (*n* = 49), found that after administering a virtual Trier Social Stress Test (TSST), participants in more enclosed virtual environments exhibited a 113.1% increase in cortisol levels compared to a 50% increase in cortisol among participants in more open rooms. Similarly, in a small study using fMRI (*n* = 18), Vartanian et al. [13] found that enclosed spaces activated the anterior midcingulate cortex (aMCC), an area associated with fear-related stress responses. Proportions, expressed through variations in ceiling height, aspect ratio, and scale, have also been shown to impact stress responses. Studies suggest that higher ceilings and larger windows are associated with reduced stress, while lower ceilings and smaller windows are linked to increased stress. Kim et al. [14] found that specific window ratios associated with ceiling height and aspect ratio affected stress levels in 33 female participants (*n* = 33) in private rooms in a postpartum care center. Similarly, in a study conducted among 112 participants (*n* = 112), Shemesh et al. [9] found that narrow spaces induced greater stress responses, as evidenced by increased pupil diameter and beta wave activity. However, proportion and enclosure are connected to a range of environmental factors that influence physiological responses and allostatic load, such as daylight exposure and reliance on artificial lighting. Some forms of artificial light have been associated with light flicker, which can cause visual discomfort, particularly in individuals with heightened sensitivity to visual stimuli or environmental stressors [38]. The interconnected nature of environmental variables and the methodological difficulties in isolating specific architectural features for quantitative analysis present significant challenges for researchers examining the neurophysiological impact of the built environment on occupants.

#### 2.1.2. Visual Processing

Perception and experience of the built environment are fundamentally shaped by the human visual system—one of the primary means of interacting with the environment [39]. The visual system, which includes the cornea, lens, retina, and neural pathways to the occipital cortex, processes visual stimuli [40]. The retina captures light and converts it into neural signals, which the brain decodes to represent the external environment [40]. There is a profound interdependence between visual information processing and physiological responses, highlighting the role of visual processing in shaping human engagement with the environment and in influencing psychological states and behaviors.

##### Daylight

Daylight is crucial for human health in regulating circadian rhythms—the natural 24 h cycles governing sleep–wake patterns, hormone release, and other physiological functions [41]. Daylight affects the nervous and endocrine systems, particularly melatonin secretion [41]. Melatonin levels peak at night in darkness, promoting sleep, and decrease during the day, enhancing alertness [42]. Disruptions caused by insufficient daylight exposure and excessive artificial light at night lead to dysregulation in internal temporal order and melatonin production, negatively impacting health [43,44].

Disruption of circadian rhythms has been implicated in heightened inflammatory responses, which are known to contribute to both metabolic and mental health disorders. Research by Sookoian et al. [45] demonstrates that circadian misalignment, such as that experienced in rotating shift work, is associated with increased biomarkers of inflammation, suggesting a direct pathway by which circadian disruptions impact systemic health. Similarly, Lyall et al. [46] identify inflammatory subtypes of depression, emphasizing the role of inflammation as a key link between circadian dysregulation and mental health conditions. This growing body of evidence supports the hypothesis that circadian rhythm disturbances exacerbate inflammation, creating a bidirectional relationship with mental health challenges, and highlights the need for further research into interventions targeting circadian alignment to mitigate these effects.

Circadian rhythm disruptions can lead to dysregulation of the HPA axis, impairing stress adaptation [47]. The circadian system, regulated by the suprachiasmatic nucleus (SCN), integrates environmental cues like light/dark cycles, synchronizing internal physiological systems by regulating allostatic mediators, including glucocorticoids and norepinephrine [48]. The circadian system is pivotal in maintaining homeostasis and as part of the body’s adaptive response to stress [47]. Misalignment between the SCN and peripheral clocks—due to limited daylight or unpredictable stressors—leads to dysregulated HPA axis activity, resulting in irregular secretion of glucocorticoids and norepinephrine [47,48]. This creates imbalances in the feedback loops that regulate the HPA axis and other signaling systems [49] and can result in accumulated allostatic load.

Architectural features like window size, surface reflectance, surrounding building heights, building and room orientation, room geometry, and glazing materials significantly affect daylight and artificial light characteristics within buildings [50,51,52,53,54]. Altenberg Vaz and Inanici [53], identified six key parameters—view direction, window head height, building orientation, shading devices, external obstructions, and room depth—as critical for access to daylight, with view direction having the most significant impact on circadian potential. In a study conducted among 112 healthy young males (*n* = 112), Petrowski et al. [55] investigated the impact of light intensity and spectral composition on cortisol stress responses. They found that exposure to bright light led to the highest cortisol levels among participants, highlighting the influence of light intensity and wavelength on cortisol production, and the potential impact of the built environment as an environmental mediator in cortisol production.

##### Light Flicker

Exposure to temporal light modulation (TLM), or “flicker”, has been recognized to affect human health. TLM, characterized by fluctuations in light output, is common in modern lighting systems such as fluorescent and LED lamps [56]. Artificial light sources often produce flicker, which can induce negative physiological and neurological responses, making it a critical consideration in built environment design [38,56,57]. Flickering light can cause headaches, eye strain, and visual disturbances [38]. Some of the neurological effects that have been noted include exacerbating conditions such as photosensitive epilepsy [58]. Chronic exposure to flickering light can lead to physiological stress that can manifest as changes in heart rate, blood pressure and visual discomfort and can contribute to long-term health issues [38,58,59]. Contemporary architecture’s increasing reliance on artificial lighting exacerbates this issue [50] and is often further compounded by the use of some energy-efficient lighting solutions such as LEDs [60], which can flicker more due to lower-quality drivers that fail to adequately regulate current.

Veitch et al. [38] studied TLM’s impact on neurophysiology, finding that 100 Hz TLM was associated with larger pupil sizes and increased ERP dipole strength, suggesting that flicker at certain frequencies can influence brain activity and arousal levels and may potentially increase cognitive interference and negatively affect reading speed. Research by Veitch and Miller [59] emphasizes TLM’s role, particularly for individuals with visual sensitivity, noting that tailoring lighting frequencies and modulation depths to reduce flicker-induced discomfort, could prevent unnecessary cognitive interference and support better task performance. The study showed that higher modulation depths at 500 Hz significantly increased cognitive load and resulted in slower response times, especially in individuals with high pattern glare sensitivity (PGS). Lighting in built environments should be tailored to accommodate different susceptibility to light sensitivity so as to prevent cognitive overload and discomfort.

#### 2.1.3. Visual Patterns

Visual patterns, particularly those that exhibit geometric and repetitive designs, have the potential to induce visual stress, leading to adverse health outcomes such as headaches, migraines, or seizures in susceptible individuals. Visual discomfort arises when the visual system encounters patterns that deviate from the spatial characteristics typically found in nature [61]. Natural images share common statistical features—namely a 1/f spatial frequency distribution where the amplitude of details diminishes as frequency increases—that facilitate efficient encoding by cortical neurons.

Deviations from these natural properties, common in modern urban environments [15,62], can induce visual discomfort and negatively affect roughly 10–15% of the population [15]. High-contrast visual patterns in architectural features can cause visual discomfort, especially at spatial frequencies of approximately three cycles per degree of visual angle [15]. These patterns can be found in some stair treads, highly patterned carpets, venetian blinds, ventilation grills, acoustic ceiling panels, and some facade paneling [63]. Le et al. [61] explored how statistical properties of visual scenes influence perception and neural processing. In analyzing images of building facades, they found that facades that deviated to a greater degree from natural statistical properties elicited a greater haemodynamic response in the primary visual cortex, as measured using functional near-infrared spectroscopy (fNIRS). Song et al. [64] investigated the physiological effects of visual stimulation using forest images versus urban images on brain activity and autonomic function in 17 female university students. They found that forest imagery led to decreased oxy-Hb concentrations in the right prefrontal cortex and increased feelings of being “comfortable”, and “relaxed”—suggesting that the forest images had a positive effect on stress reduction.

### 2.2. From Adaptation to Maladaptation: Allostatic Load and Overload

Allostatic processes are the physiological responses that the body initiates in reaction to stress-inducing events, to restore equilibrium or homeostasis. These processes are essential for regulating physiological functions over time and mitigating the harmful impacts of stressors. In this context, a stressor is defined as a “threat, real or implied, to the psychological or physiological integrity of an individual” ([65], p. 108). Research indicates that when the body is able to rapidly downregulate after a stress response, these allostatic mechanisms serve a protective and beneficial role in the body’s physiological response to stress.

While acute stress responses play a crucial protective role for survival, prolonged exposure to stress can cause these initially adaptive physiological mechanisms to become maladaptive. This shift contributes to allostatic load—the cumulative physiological burden on the body’s regulatory systems that results from sustained activation of stress responses [65]. Allostatic load represents the “wear and tear” on the body and brain resulting from chronic overactivation, or underactivation, of physiological stress response systems [65].

The progressive accumulation of stress-related dysregulation can eventually lead to a pathological state known as allostatic overload [66]. Allostatic overload occurs when the body’s adaptive mechanisms for maintaining homeostasis—collectively termed allostasis—are overtaxed due to chronic exposure to stressors [66]. In this state, the allostatic systems—including the hypothalamic–pituitary–adrenal (HPA) axis, the autonomic nervous system, the metabolic system, and the immune system—are chronically activated or fail to shut down appropriately after stress exposure [67]. This persistent activation leads to a cascade of deleterious physiological events [67].

Chronic stress results in sustained secretion of stress hormones such as cortisol and catecholamines (epinephrine and norepinephrine) [68]. Elevated cortisol levels can cause hippocampal atrophy and impaired learning and memory [69]. The prefrontal cortex, essential for executive functions, can also be adversely affected, leading to impaired decision-making and emotional regulation [70]. Additionally, elevated glucocorticoids suppress immune function by inhibiting the proliferation of lymphocytes and reducing the production of pro-inflammatory cytokines [71]. While acute stress can enhance immune readiness, chronic stress can lead to immunosuppression, increased susceptibility to infections, and slower healing rates [72]. Paradoxically, chronic stress can also promote a pro-inflammatory state due to glucocorticoid receptor resistance, in which immune cells become less sensitive to cortisol’s anti-inflammatory effects [73]. This leads to elevated levels of inflammatory markers like C-reactive protein (CRP) and interleukin-6 (IL-6) and can contribute to the development of a range of chronic diseases [74].

Chronic stress has widespread impacts on both biological and neurological processes. One major mechanism is the shortening of telomeres, a marker of biological aging, which is associated with an increased risk of age-related diseases and reduced lifespan [75]. Chronic stress may also influence the onset and symptom severity of many mental health conditions [76]. Additionally, chronic exposure to stress hormones adversely impacts the brain by inhibiting neurogenesis in the hippocampus and reducing synaptic plasticity, which not only impairs cognitive functions but also diminishes resilience to stress [77]. Together, these interconnected effects illustrate the profound and multifaceted impacts of chronic stress on both the body and mind.

Allostatic overload represents a tipping point where the cumulative burden of chronic stress overwhelms the body’s physiological adaptive mechanisms, transforming adaptive processes into maladaptive outcomes [66]. This state not only compromises immediate physiological functioning but also sets the stage for the potential development of a multitude of stress-related diseases. It highlights the critical importance of effective stress management and intervention strategies to prevent a range of potential long-term health consequences associated with chronic stress.

### 2.3. Implications of the Built Environment on Allostatic Overload

In the context of the built environment, the allostatic responses triggered by exposure to stress-inducing built environment elements may appear harmless or within tolerable limits when experienced as discrete acute events. However, the likelihood of negative health outcomes resulting from these processes is significantly influenced by the intensity and duration of exposure. Built environments per se are not inherently detrimental to human health; the primary concern arises from the fact that stress-inducing built environment elements are seldom unique nor isolated exposure occurrences. Stress-inducing environments are ubiquitous and form part of everyday spaces such as workplaces, homes, hospitals, and educational institutions. Consequently, architectural features such as rooms with little-to-no natural light, spaces with high-contrast repeat striations, soft furnishings that exhibit certain patterns, or confined spaces with poor airflow—which may seem tolerable in isolation as a discreet exposure event—could have detrimental health effects when experienced over prolonged periods.

A pressing issue is the increasing exposure to the built environment on a global scale. By 2050, projections indicate that 70% of the world’s population will reside in urban areas [78]. On average, individuals in developed countries spend over 90% of their time indoors [79]. Vulnerable groups—such as the elderly, infants and young children, and those with compromised immune systems—may spend up to 100% of their time indoors [80].

Despite these findings and research on the physiological impacts of discrete exposure to specific built environment conditions, current research has not clearly established the precise impact of extended or repeated exposure to stress-inducing architectural forms on human health, nor whether such exposure leads to allostatic overload. The primary concern is that the cumulative effect of these environmental stressors could contribute to chronic stress, potentially pushing individuals toward allostatic overload. Given the significant health implications associated with allostatic overload, it is crucial to investigate the role of architectural design in either mitigating or exacerbating stress responses.

## 3. Allostatic Overload and Neurosustainability

To date, research on the impact of the built environment on allostatic overload has largely focused on neuroimmunological activity and inflammation [81]. However, the emerging concept of neurosustainability [18] highlights the critical role of naturally enriched environments in sustaining neuroplasticity processes and sheds light on the potential adverse effects of architecturally mediated allostatic overload. Chronic stress and elevated allostatic load not only affect physical health but also have significant implications for brain function in relation to the adverse impact on neuroplasticity.

### 3.1. Neurosustainability

Neurosustainability is the maintenance of adaptive neuroplasticity through environmental enrichment mechanisms innately found in nature [18]. Environmental enrichment is well established in animal models, and its mechanisms are a triangulation of physical activity, cognitive stimulation, and homeostasis [17,82,83,84]. For instance, in natural environments, greenness (in the form of tree cover density, etc.) has been found to be an effective modulator of positive neuroplasticity across the three brain regions (amygdala, hippocampus, and cortex) [85,86,87,88]. However, to date, there is little understanding of the extent to which the built environment can be considered as enriching [89], and there is little evidence to suggest that the design of the built environment, besides greenness, can approximate the effects of the natural environment on neuroplasticity [90]. The adverse impact of elements found within the built environment can not only inhibit the sustainability of neuroplasticity processes but can lead to negative neuroplasticity in situations that result in allostatic load [91,92]. To this end, it is important to understand the adverse effects of architecturally mediated allostatic overload so as to better understand how to shape the design of the built environment for neurosustainability and maximize the benefits of exposure to enriched environments.

### 3.2. Stress and Inhibited Neurosustainability Through HPA Axis Activation

Allostasis and homeostasis can affect the sustainability of the neuroplasticity of various brain regions such as, but not limited to, the cortex, hippocampus, and the amygdala. Homeostasis, a term first coined by Walter Cannon [93], refers to the maintenance of a state of internal balance through physiological regulation and constant adjustments to bodily systems, while allostasis emphasizes the dynamic regulatory processes that prepare the body for anticipated challenges [94]. Both are vital to normal physiological functioning; however, chronic allostatic load can have profound implications on brain structures such as the amygdala, hippocampus, and cortex through the dysregulation of the HPA axis.

The amygdala is highly responsive to stress and plays a pivotal role in identifying and responding to threats—both real and perceived. In response to stressors, the amygdala serves to trigger autonomic responses by activating the HPA axis stress response system [95,96,97,98]. In a recent study examining the impact of natural environments on the reactivity of the amygdala to stress (*n* = 63), it was found that the amygdala’s reactivity to stress-inducing stimuli was reduced after a 1 h walk in a natural environment. However, this reduction in the stress response was only observed among female participants when exposed to natural environments, and not when exposed to built environments [86,87]. Similarly, a study by Harris et al. [99] found that exposure to gray space (i.e., impervious surfaces such as concrete, streets, or rooftops) in the built environment increases the risk of emotional dysregulation, as observed in the amygdala–DMN connectivity—circuits that have been implicated in psychopathology. As can be seen from these studies, not only is the amygdala’s functional plasticity positively responsive to green environmental features (i.e., vegetation and natural landscapes), but environment variables such as the presence of gray built environment features such as concrete can cause adverse plasticity outcomes in the amygdala and may contribute to architecturally mediated allostatic overload. Furthermore, amygdala activity can significantly affect memory consolidation and neural plasticity in other brain regions such as the hippocampus, and the same amygdala mechanisms that facilitate the robust encoding of emotionally salient memories can be maladaptive under conditions of chronic stress [100]. The findings from research to date highlight the need for further research to fully explore the impact of the environmental milieu on the interrelationship between the amygdala and stress responses [86,87]. This leads to the second key brain region, the hippocampus.

The hippocampus is involved in both short-term and long-term stress responses. Evidence suggests that exposure to natural environments following acute stress can foster adaptive structural plasticity, specifically increasing bilateral subiculum volume [88]. As the subiculum contributes to inhibiting the stress-associated HPA axis [100,101,102,103,104], its stress-inhibitory function and the hippocampus’s overall sensitivity to acute stressors highlight the importance of examining how architecturally mediated allostatic overload may adversely influence the immediate structural plasticity of the hippocampal subiculum. Stress-induced hippocampal impairment is known to result in negative changes in learning and memory function [105]. This can result in long-term neurological changes due to the regulation of adult hippocampal neurogenesis in humans [106]. A possible way to counteract stress is through physical activity [107], and there is a need for research that examines environmental affordances for physical activity that stimulates the release of the brain-derived neurotrophic factor (BDNF) that is responsible for regulating adaptive neuroplasticity [18,19,108,109].

Hippocampal neurogenesis is one of the neuroplasticity processes most affected by stress. Stress leads to a rapid and prolonged decrease in the rate of cell proliferation [110]. Stress hormones have potent growth-inhibiting effects that ultimately hinder neurogenesis in the hippocampus [111]. Glucocorticoids and other stress-related biomolecules have been associated with impaired neurogenesis [112,113,114]. At the molecular level, several pathways that affect neurogenesis are also modulated by stress, such as the production of cytokines and neurotrophic factors, morphogen signaling pathways, and the synthesis of glucocorticoid [115]. In a review by Saaltink and Vreugdenhil [116], the role of the glucocorticoid receptor in the complex interaction between stress and adult neurogenesis was examined. The glucocorticoid receptor is the primary mediator of the stress response—from proliferation to differentiation, migration, and functional integration. Saaltink and Vreugdenhil [116] posit that an excitation–inhibition imbalance underlies the association between aberrant integration of newborn neurons and psychiatric and paroxysmal brain disorders. The relationship between hippocampal neurogenesis, stress, and antidepressants has long been explored [117,118]. Dysregulated stress responses, neuroinflammation, inhibited neurogenesis, and synaptic plasticity imbalances have all been found to be present in the molecular and neurophysiological etiology of depression [119]. Furthermore, Schoenfeld et al. [120] found that chronic unpredictable restraint exposure to stress over 4 weeks decreased total hippocampal volume—supporting the hypothesis that stress induced by architectural allostasis can have adverse effects on hippocampal plasticity. Evaluating how architectural design might alleviate allostatic overload could be instrumental in optimizing the benefits of adult hippocampal neurogenesis.

Behavioral stress also affects both the structure and function of the prefrontal cortex [121]. Developmental stressors modify the organization of the prefrontal cortex in adulthood, with the degree to which this occurs varying based on age [122]. These stress-induced structural changes impact top-down executive functions such as decision-making processes that are facilitated by the prefrontal cortex [123]. In a study by Misquitta et al. [124], chronic stress was found to be associated with reduced anterior cingulate cortex volume. This effect has been found to be particularly notable in individuals with post-traumatic stress disorder [113]. Research suggests that stress may have a long-term impact on the plasticity of the cerebral cortex, but even short-term (acute) stress can influence the functioning of important areas like the prefrontal cortex.

### 3.3. Architecturally Mediated Allostasis, HPA Axis Activation, and Impaired Neurosustainability

Neurophysiological evidence has established a link between stress exposure and maladaptive neuroplasticity, underscoring the necessity to deepen our understanding of how the built environment and environmental enrichment mechanisms influence neuroplasticity and, in turn, neurosustainability. Built environment and visual elements that induce allostatic overload can adversely affect neuroplasticity at multiple levels. Considering that many individuals spend the majority of their time within built environments and are subject to chronic exposure to a range of stress-inducing environmental stimuli, the cumulative impact on neuroplasticity becomes particularly significant [79]. Acute stressors generated by the built environment may have immediate effects on both the amygdala and the hippocampal subiculum through the activation of the hypothalamic–pituitary–adrenal (HPA) axis [125]. Research by Steinheuser et al. [126] demonstrated that urban upbringing alters the reactivity of the HPA axis. These structural changes may be attributed, in part, to architecturally mediated allostatic overload and the subsequent alterations in the structural plasticity of both the amygdala and hippocampal subiculum in response to acute stressors. Consequently, there is a critical need to examine how design interventions within the built environment may mitigate architecturally mediated stress and support neural sustainability. Of particular interest is the potential for the incorporation of natural elements with salutogenic effects into architectural and visual forms, as well as the development of design strategies that optimize neurosustainability and facilitate the effective adaptive regulation of neuroplasticity to reduce the onset of chronic allostatic overload. Table 1, Table 2, Table 3 and Table 4 provide a comprehensive synthesis of the research on key relationships between the built environment and allostatic overload.

## 4. Discussion

The adverse effects of architecturally mediated allostasis on stress are significant. Chronic exposure to stress-inducing environments undermines neurosustainability by disrupting the internal homeostasis that regulates neuroplasticity and essential biomarkers. Architecturally mediated allostasis encompasses environments that are not only lacking in enrichment but also those that actively induce stress responses. These environments fail to provide the necessary elements of environmental enrichment that are crucial for maintaining neural health and resilience while simultaneously triggering physiological stress pathways [127]. Such conditions may alter the body’s stress response systems, potentially influencing brain structure and function.

**Figure 3:**
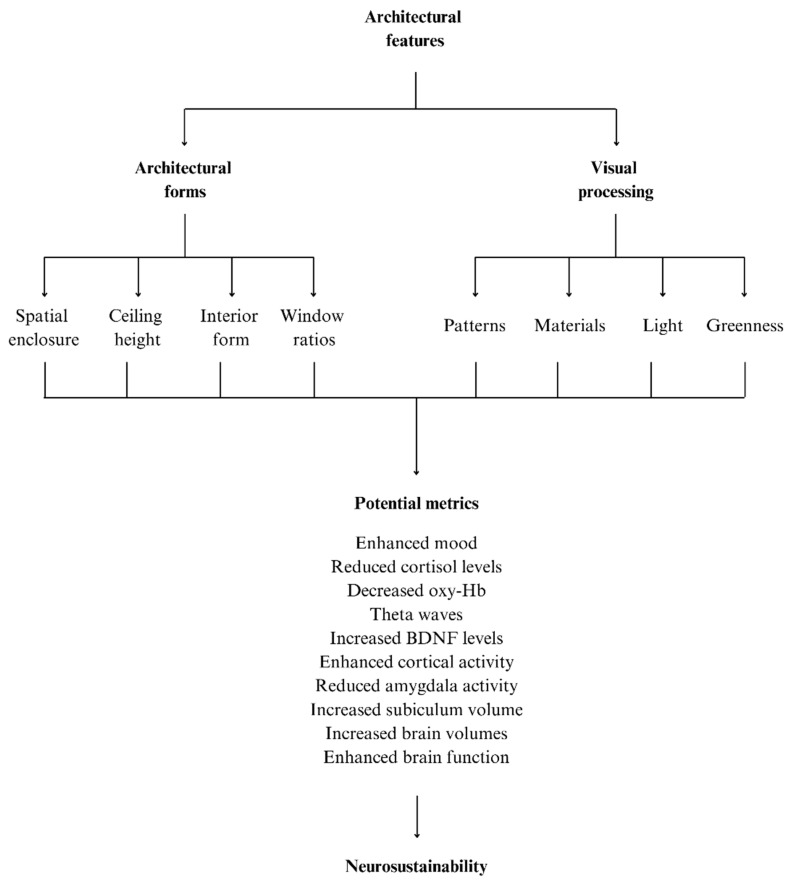
A framework for potential avenues for future research examining the impact of architectural stressors on neurosustainability.

The theoretical framework presented in this paper provides a foundational model linking the design of the built environment to neurocognitive health. However, as a conceptual model, it necessitates empirical testing to validate and refine the conceptual framework outlined within this paper. Future research should adopt a multifaceted approach, integrating clinical studies, neurogenesis investigations, and longitudinal environmental epidemiology to comprehensively assess the framework’s validity and applicability. To empirically test the proposed framework, initial empirical studies may focus on examining neurophysiological responses to various architectural stimuli, such as the presence of biophilic design elements, affordances for physical movement within the built environment, natural light, spatial enclosure, potential visual stressors, and a range of other design elements (see Figure 3). In utilizing advanced neuroimaging techniques such as functional magnetic resonance imaging (fMRI), Electroencephalography (EEG), and functional near-infrared spectroscopy (fNIRS), researchers can assess brain activity patterns in response to different architectural elements, such as curvature, lighting, and spatial layout. Additionally, there are a range of physiological biomarkers, including cortisol levels, heart rate variability (HRV), and galvanic skin response (GSR), that are able to be measured to quantify stress responses elicited by specific architectural designs. Given the complexity in examining the impact of specific aspects of the built environment on neurophysiological responses, the presence of confounding variables, and the potential subjectivity of self-reported survey based metrics, controlled studies that utilize a range of metrics and tools that enable a multi-modal approach may provide more robust means through which to examine the impact of the built environment on neurogenesis and neurophysiological wellbeing [128]. For instance, controlled experiments could expose participants to environments with varying degrees of enclosure and curvature to observe corresponding changes in the HPA and SAM axis activities, while incorporating standardized clinimetric assessments—systematic evaluations used to measure clinical symptoms and patient functioning—to capture subjective stress experiences [78]. Such studies have the potential to provide direct evidence of the ways in which architectural features impact neurophysiological stress mechanisms through both objective biomarkers and validated clinimetric tools and provide much-needed insights into the impact of architecturally mediated allostasis on neurosustainability.

Given the complexity of the interactions between the built environment and neurocognitive health, future research needs to adopt an interdisciplinary and multi-modal approach, bridging neuroscience, architecture, psychology, and public health [128]. For instance, evidence suggests that synchronous architecturally mediated impairment of the amygdala, hippocampus, and cortex may play a role in the neural underpinnings of borderline personality disorder; developing a better understanding of the built environment factors that contribute to conditions such as borderline personality disorder has the potential to have profound public health ramifications [129]. Collaborative efforts between architects and neuroscientists can facilitate the design of studies that accurately reflect real-world architectural variations while employing rigorous scientific methodologies. Furthermore, integrating qualitative approaches, such as participant interviews and ethnographic studies, can provide deeper insights into the subjective experiences of individuals within different built environments, complementing quantitative findings. Translational research efforts offer the potential to transform empirical findings into evidence-based design principles that can be implemented in built environment projects—from private residential to commercial, educational, and public spaces. Through interdisciplinary collaboration and longitudinal investigation, future studies have the potential to inform and support architectural innovations that enhance neurocognitive health and overall wellbeing.

## 5. Conclusions

The emerging body of research examined in this paper underscores the critical importance of recognizing the built environment as both a potential mediator and moderator of neurocognitive health and wellbeing. By integrating insights from neuroscience, environmental psychology, physiology, and built environment design, this paper advances a theoretical framework that situates built environment design as an influential factor in shaping neurophysiological stress responses. Specifically, this paper highlights how features of the built environment—such as curvature, enclosure, visual complexity, and lighting conditions—can directly and indirectly influence the balance between homeostatic and allostatic mechanisms, potentially facilitating or impairing neuroplastic changes and affecting long-term health outcomes, including the risk of mood disorders and cognitive impairments in the case of allostasis.

The concept of architecturally mediated allostasis posits that chronic exposure to stress-inducing built environments may transition from adaptive stress responses to maladaptive patterns of allostatic overload. This sustained physiological burden can disrupt neuroplastic processes in key brain regions including the amygdala, hippocampus, and cortex, hindering neurosustainability—the maintenance and enhancement of healthy brain function via environmental enrichment. While the detrimental consequences of stress-induced neurobiological changes are profound, these findings also point toward the potential for actionable opportunities to improve wellbeing. Built environment interventions that incorporate evidence-based design principles—such as increasing natural light, reducing visual light flicker, employing biophilic design elements, optimizing spatial layouts, and mindful material selections—have the potential to diminish physiological stress reactivity, support adaptive neuroplasticity, and thereby nurture mental wellbeing and mental and cognitive health.

The trajectory of future research in this interdisciplinary field will benefit from more robust, longitudinal, and controlled studies, as well as advancements in measurement methodologies—ranging from VR simulations and biometric indicators to imaging techniques—to clarify causal pathways. Such endeavors may serve to help inform guidelines and best practices for architects, urban planners, healthcare professionals, and policymakers that can help shape design for better neurophysiological health. By fostering environments that buffer stress, enhance cognitive functioning, and sustain neural health, we set the stage for built environments that are not only visually and functionally appealing but also fundamentally health-promoting. This paradigm shift in how we conceive and design our spaces represents a transformative step forward in the pursuit of urban and architectural design interventions that support neurophysiological wellbeing, mental health, and resilience.

## Figures and Tables

**Table 1:** Comprehensive research synthesis of built environment effects on neurophysiology and allostatic load: direct built environment effects.

Study	Architectural/ Environmental Factor	Neurophysiological Finding	Impact on Allostatic Load/Neurosustainability
Steinheuser et al. (2014) [126]	Urban upbringing	Altered HPA axis reactivity	Early urban exposure permanently alters stress response systems
Fich et al. (2014) [11]	Spatial enclosure	113.1% increase in cortisol in enclosed spaces vs. 50% in open spaces	Direct relationship between architectural features and HPA axis activation
Vartanian et al. (2015) [13]	Ceiling height and enclosure	Activation of anterior midcingulate cortex	Neural correlates of architectural features related to stress
Banaei et al. (2017) [12]	Interior forms/curvature	Enhanced pleasure and emotional arousal (increased theta wave activity)	Architectural form directly influences brain activity
Kim et al. (2021) [14]	Window ratios and ceiling height	Changes in stress levels based on proportions	Spatial proportions affect physiological responses
Shemesh et al. (2021) [36]	Spatial geometry	Increased pupil diameter and beta wave activity in narrow spaces	Spatial constraints impact physiological state
Shemesh et al. (2022) [9]	Geometric forms in virtual spaces	Inverse relationship between wall curvature and stress	Specific design elements modulate stress responses
Li et al. (2022) [35]	Curved transitional spaces	Enhanced stress recovery after TSST exposure	Certain forms offer stress-buffering effects
Strachan-Regan and Baumann (2024) [37]	Interior forms/curvature	Lower heart rate in curved rooms/increased positive affect, reduced negative affect	Room geometry influences physiological arousal

**Table 2:** Comprehensive Research synthesis of built environment effects on neurophysiology and allostatic load: biophilic elements and comparative studies.

Study	Architectural/ Environmental Factor	Neurophysiological Finding	Impact on Allostatic Load/Neurosustainability
Song et al. (2018) [64]	Forest vs. urban imagery	Decreased oxy-Hb concentrations in prefrontal cortex	Visual natural elements reduce neural stress markers
Kühn et al. (2021) [85]	Urban green space	Changes in brain structure	Long-term neuroplastic adaptations to environmental features
Sudimac and Kühn (2022) [86,87]	Natural vs. built environment	Reduced amygdala activity after nature exposure (in women)	Built environments may maintain elevated stress responses
Harris et al. (2023) [99]	Gray space exposure	Altered amygdala–DMN connectivity	Built environment features affect emotional regulation circuits
Sudimac and Kühn (2024) [88]	Forest exposure	Changes in hippocampal plasticity	Natural elements promote positive neuroplasticity

**Table 3:** Comprehensive research synthesis of built environment effects on neurophysiology and allostatic load: light and circadian effects.

Study	Architectural/ Environmental Factor	Neurophysiological Finding	Impact on Allostatic Load/Neurosustainability
Sookoian et al. (2007) [45]	Circadian disruption	Increased inflammatory biomarkers	Environmental conditions influence systemic inflammation
Lyall et al. (2018) [46]	Circadian disruption	Altered mood and cognitive function	Built environment impacts biological rhythms
Petrowski et al. (2021) [55]	Light exposure variations	Different cortisol responses based on light intensity	Lighting decisions impact stress hormone production
Veitch et al. (2024) [38]	Temporal light modulation	Changes in cognitive performance and brain function	Lighting design affects neurophysiological function

**Table 4:** Comprehensive Research synthesis of built environment effects on neurophysiology and allostatic load: neuroplasticity and chronic stress.

Study	Architectural/ Environmental Factor	Neurophysiological Finding	Impact on Allostatic Load/Neurosustainability
Warner-Schmidt and Duman (2006) [110]	Environmental stress	Hippocampal neurogenesis inhibition	Stress impairs neural regeneration
Saaltink and Vreugdenhil (2014) [116]	Stress response mechanisms	Glucocorticoid receptor regulation of neurogenesis	Stress affects adult neurogenesis processes
Schoenfeld et al. (2017) [120]	Chronic stress exposure	Decreased hippocampal volume	Prolonged stress impacts brain structure
Misquitta et al. (2021) [124]	Chronic stress exposure	Reduced anterior cingulate cortex volume	Sustained stress causes structural brain changes
Khalil (2024) [108]	Built environment affordances	Physical activity promotion through design	Environmental design can support neurosustainability

## Data Availability

No new data were created or analyzed in this study. Data sharing is not applicable to this article.

## Abbreviations

ACC	Anterior cingulate cortex
ACTH	Adrenocorticotropic hormone
aMCC	Anterior midcingulate cortex
AVP	Arginine vasopressin
BDNF	Brain-derived neurotrophic factor
CRH	Corticotropin-releasing hormone
CRP	C-reactive protein
DMN	Default mode network
EEG	Electroencephalography
ERP	Event-related potential
fMRI	Functional magnetic resonance imaging
fNIRS	Functional near-infrared spectroscopy
GSR	Galvanic skin response
HPA	Hypothalamic–pituitary–adrenal (axis)
HRV	Heart rate variability
IL-6	Interleukin-6
LED	Light-emitting diode
MET	Metabolic equivalent (commonly used as METs)
PFC	Prefrontal cortex
PGS	Pattern glare sensitivity
PVN	Paraventricular nucleus (of the hypothalamus)
SAM	Sympathetic–adreno-medullary (axis)
SCN	Suprachiasmatic nucleus
TLM	Temporal light modulation
TSST	Trier Social Stress Test
UN	United Nations
VR	Virtual reality
WHO	World Health Organization
